# Ectopic expression of BpbHLH9 suggested the presence of a self-activating loop mechanism of clade Ia bHLHs to enhance betulinic acid biosynthesis in *Lotus japonicus* hairy roots

**DOI:** 10.5511/plantbiotechnology.24.0717b

**Published:** 2024-09-25

**Authors:** Hayato Suzuki, Shigeo S. Sugano, Toshiya Muranaka, Hikaru Seki

**Affiliations:** 1 Plant Molecular Technology Research Group, Bioproduction Research Institute, National Institute of Advanced Industrial Science and Technology (AIST), Sapporo, Hokkaido 062-8517, Japan; 2 Plant Gene Regulation Research Group, Bioproduction Research Institute, National Institute of Advanced Industrial Science and Technology (AIST), Tsukuba, Ibaraki 305-8566, Japan; 3 Department of Biotechnology, Graduate School of Engineering, Osaka University, Suita, Osaka 565-0871, Japan; 4 Industrial Biotechnology Initiative Division, Institute for Open and Transdisciplinary Research Initiatives, Osaka University, Suita, Osaka 565-0871, Japan

**Keywords:** betulinic acid, bHLH transcription factor, hairy root, RNA-sequencing, triterpene

## Abstract

For the optimal production of specialized (secondary) metabolites in plant hosts, a comprehensive understanding of their regulatory mechanisms is imperative. Bioactive C-28-oxidized triterpenes, such as oleanolic, ursolic, and betulinic acids, are metabolites ubiquitously found across the plant kingdom; however the precise regulatory mechanisms governing their biosynthesis remain elusive. Previously, we demonstrated that the clade Ia bHLH transcription factor, LjbHLH50, plays a pivotal role in the upregulation of betulinic acid biosynthesis in *Lotus japonicus*. However, inconsistent outcomes have been observed in transient effector-reporter assays, which are commonly employed in transcription factor studies. Thus, in the present study, we sought to further characterize LjbHLH50 by examining the ectopic expression of *BpbHLH9*, a homolog of *LjbHLH50* in *Betula platyphylla*, in *L. japonicus* hairy roots. Remarkably, *BpbHLH9* expression elicited metabolic and transcriptomic alterations almost similar to those induced by *LjbHLH50* overexpression, highlighting the conserved function of clade Ia bHLHs. Through RNA-sequencing analysis, we found that *LjbHLH50* was upregulated by ectopic BpbHLH9 expression, implying the existence of a self-activating loop in clade Ia bHLHs that facilitates enhanced betulinic acid biosynthesis. Notably, among the clade Ia bHLHs homologous to *BpbHLH9*, *LjbHLH50* and two *LjbHLH50* paralogs were upregulated upon BpbHLH9 induction, underscoring the central role of these clade Ia bHLHs in betulinic acid biosynthesis regulatory networks in *L. japonicus* hairy roots.

C-28-oxidized triterpenes, namely oleanolic (OA), ursolic (UA), and betulinic acids (BA) exhibit useful bioactivities in humans, such as antifungal, antibacterial, anti-HIV, and antitumor activities (Fulda and Kroemer 2009; Perez 2003; Wu 2009). They are also important precursors of highly modified triterpene saponins, such as the vaccine adjuvant QS-21 in *Quillaja saponaria* (Martin et al. 2024), the major bioactive compound asiaticoside in the Ayurvedic and Chinese medicinal plant *Centella asiatica* (Bandopadhyay et al. 2023), and bitter saponins in the pseudocereal *Chenopodium quinoa* (Meyer et al. 1990).

Triterpenes and triterpene saponins are biosynthesized from the common precursor 2,3-oxidosqualene through multiple enzymatic steps catalyzed by oxidosqualene cyclases (OSCs), cytochrome P450 monooxygenases (P450s), UDP sugar-dependent glycosyltransferases (UGTs) and others (Thimmappa et al. 2014). The CYP716A subfamily of P450s are multifunctional enzymes that catalyze the three-step oxidation of the C-28 positions of some triterpene scaffolds to produce C-28-oxidized triterpenes. Although CYP716A and C-28-oxidized triterpenes are widely distributed in the plant kingdom, their biosynthetic regulatory mechanisms are poorly understood.

Several transcription factors (TFs) regulating triterpene biosynthesis have been identified in the basic-helix-loop-helix (bHLH) family, which is one of the largest TF superfamilies in plants. The bHLH TFs were categorized into approximately 25 clades based on their sequence similarity and domain structure (Heim et al. 2003). To date, two clade Ia bHLHs have been recognized as regulatory factors for BA and OA biosynthesis (Supplementary Figure S1): BpbHLH9 in the Betulaceae plant *Betula platyphylla* (Yin et al. 2017) and LjbHLH50 in the Fabaceae plant *Lotus japonicus* (Suzuki et al. 2022). Despite conducting transient effector-reporter assays using Arabidopsis mesophyll cell protoplasts, LjbHLH50 failed to transactivate any promoter of the triterpene biosynthetic genes (Suzuki et al. 2022), suggesting indirect regulation or the lack of a counterpart TF in the assay. Previous study has suggested that LjbHLH50 overexpression affects the expression of various TFs which are the potential downstream TFs in the transcriptional cascade (Suzuki et al. 2022). However, RNA-sequencing (RNA-seq) results generally contain pseudo positive genes which makes time-consuming to functionally characterize candidate genes. Since the homolog *BpbHLH9* was expected to have equivalent functions to *LjbHLH50*, we performed RNA-seq of *BpbHLH9*-expressing hairy roots to predict the target TF genes of clade Ia bHLHs more precisely. As a result, ectopic *BpbHLH9* expression revealed some candidate TFs and suggested the presence of a self-activating loop of clade Ia bHLHs that sustains the expression of BA biosynthetic genes.

Fragments of *BpbHLH9* (KX518840; Supplementary Table S1) were synthesized using by gBlocks Gene Fragments (Integrated DNA Technologies, Coralville, IA). These fragments were assembled and integrated into pENTR1A (Thermo Fisher Scientific, Waltham, MA) linearized by PCR (primers 1 and 2 in Supplementary Table S2) using NEBuilder HiFi DNA Assembly Master Mix (New England Biolabs, Ipswich, MA) to construct pENTR1A-*BpbHLH9*. The expression vector pSD11-*BpbHLH9* (driving *BpbHLH9* and *GFP* expression by two independent *L. japonicus* *UBQ1* promoters) for hairy root induction was generated using Gateway LR Clonase II Enzyme Mix (Thermo Fisher Scientific, Waltham, MA). We performed hairy root transformation as described in our previous report (Suzuki et al. 2022). Briefly, *Agrobacterium rhizogenes* ATCC15834 strains harboring pSD11-*GFP* (negative control) and pSD11-*BpbHLH9* were used to infect the hypocotyls of *L. japonicus* young seedlings.

The metabolite analysis of the established lines was performed as previously described (Suzuki et al. 2019). In brief, triterpenes and saponins were extracted using methanol and acid-hydrolyzed to remove the sugar moieties of the saponins. The extracts were derivatized by trimethylsilylation and analyzed by gas chromatography-mass spectrometry (GC-MS). Their peaks were identified based on the retention time and mass fragmentation patterns of the standard pure compounds (Suzuki et al. 2019). We established each 3 hairy root line expressing GFP and BpbHLH9, respectively. *L. japonicus* hairy roots competitively produce triterpenes, such as soyasapogenol B (SB), OA, and BA. The accumulation of BA was enhanced more than that of SB and OA in the BpbHLH9-expressing lines (Figure 1).

**Figure 1:**
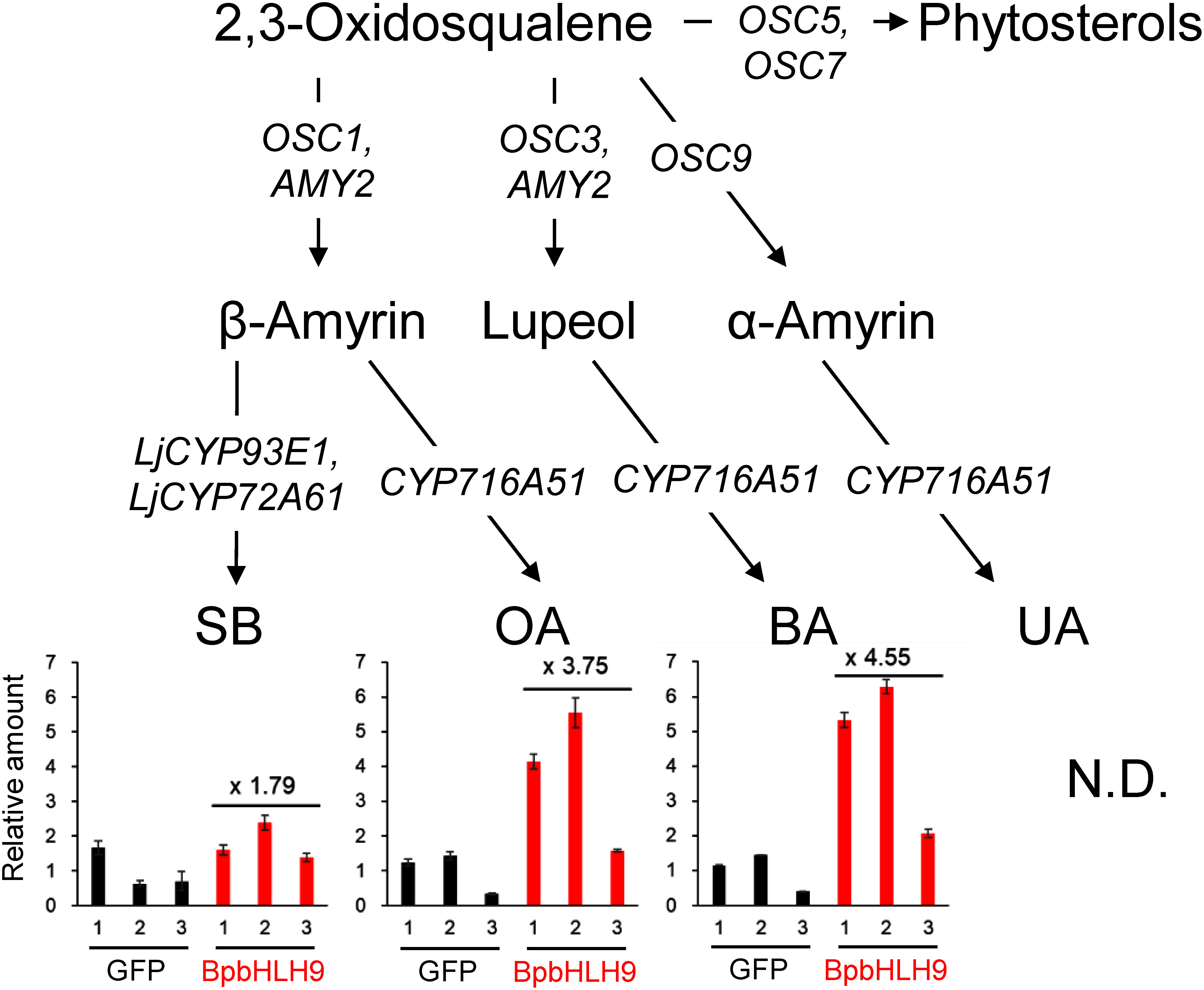
Figure 1. Analysis of GFP- and BpbHLH9-expressing *L. japonicus* hairy roots. Relative amount of soyasapogenol B (SB), oleanolic acid (OA), and betulinic acid (BA) in each hairy root line calculated from peak area of gas chromatography-mass spectrometry (GC-MS) chromatograms. The relative amount was calculated by dividing the peak area of each sample by the average peak area obtained from the three GFP expression control lines. The values on the graph were calculated using these average values.

We conducted RNA-seq analysis of hairy root lines to observe the transcriptomic changes induced by ectopic BpbHLH9 expression. Total RNA was extracted from fresh hairy roots frozen in liquid nitrogen, followed by DNase I treatment, column purification, and cDNA synthesis as described previously (Suzuki et al. 2019). Messenger RNA was purified from 1 µg of total RNA following previous study (Townsley et al. 2015). Complementary DNA libraries were prepared using a KAPA Stranded RNA-Seq Library Preparation Kit (Kapa Biosystems, Wilmington, MA) according to the manufacturer’s instructions. The libraries were sequenced using the Hiseq X-ten platform (Illumina, San Diego, CA) with 150 bp paired-end (PE) reads by Macrogen Japan (Koto-ku, Tokyo, Japan). The raw RNA-seq reads (19–27 million reads per line; Supplementary Table S3) were deposited in the DDBJ Sequence Read Archive (DRA) under the accession number DRA018328. The fastq files were processed with the quality trimming software fastp (Chen et al. 2018), followed by mapping to the *L. japonicus* genome, which was obtained from the LotusBase website (*Lotus japonicus* Gifu v1.2 assembly; https://lotus.au.dk/ (Accessed Apr 4, 2024); Kamal et al. 2020), using STAR software (Dobin et al. 2013). To calculate *BpbHLH9* expression level, *BpbHLH9* CDS sequence was included in the genome sequence file. The read counts for each gene were estimated using featureCounts (Liao et al. 2014). We employed a graphical user interface for TCC (TCC-GUI; Su et al. 2019) to identify differentially expressed genes (DEGs). Low count genes (0 mapped reads) were filtered from the TCC computation.

Principal component analysis revealed that the GFP- and BpbHLH9-expressing lines were separated in the PC1 direction (Supplementary Figure S2A). We identified 1151 upregulated DEGs (upDEGs) and 786 downregulated DEGs (downDEGs) in BpbHLH9-expressing lines compared to GFP-expressing control lines (Supplementary Figure S2B, Supplementary Table S4). We retrieved changes in the expression of triterpene-related genes from the transcriptome data (Table 1). The expression of SB biosynthetic genes (*OSC1* and *LjCYP93E1*) was not enhanced, but BA biosynthetic genes (*OSC3* and *CYP716A51*) were upregulated upon BpbHLH9 expression. AMY2, a multifunctional OSC that produces both β-amyrin and lupeol, was also upregulated. This likely contributes to the increase in OA and SB accumulation in the BpbHLH9-expressing lines (Figure 1). *CYP88D4*, an *AMY2* gene cluster member, was also upregulated by BpbHLH9 expression. However, CYP88D4 did not oxidize triterpene scaffolds in previous studies (Krokida et al. 2013; Seki et al. 2008) and the involvement of CYP88D4 in triterpene biosynthesis remains unclear. We also found the commonly upregulated transcription factor genes in *LjbHLH50*- and *BpbHLH9*-expressing hairy roots, which are promising candidates regulating BA biosynthetic genes in *L. japonicus* (Supplementary Table S5).

**Table 1:** Table 1. Expression of triterpene-related genes in transgenic *L. japonicus* hairy roots.

Gene	Gene ID	Log_2_ (Average expression level)	Log_2_ (Fold change)	FDR
Triterpene biosynthesis pathway				
OSC1 (bAS)	LotjaGi3g1v0340000	11.66	−0.51	0.15
**OSC3 (LUS)**	**LotjaGi2g1v0235400**	**8.77**	**4.15**	**6.0E−09**
OSC9 (aAS)	LotjaGi3g1v0537700_LCC	N. D.	—	—
OSC5 (CAS)	LotjaGi2g1v0235200	9.49	−0.11	0.88
OSC7 (LAS)	LotjaGi2g1v0235000	6.47	0.43	0.55
**CYP716A51**	**LotjaGi4g1v0438900**	**10.02**	**3.46**	**1.4E−16**
LjCYP93E1	LotjaGi1g1v0588600	13.89	−0.38	0.32
LjCYP72A61	LotjaGi3g1v0557600	13.41	−0.29	0.38
CSyGT	LotjaGi3g1v0336500	12.10	−0.12	0.92
Putative DDMPT	LotjaGi3g1v0557700	10.47	−0.11	0.93
AMY2 gene cluster				
**AMY2 (mix AS)/OSC8**	**LotjaGi3g1v0338300**	**8.04**	**1.51**	**2.0E−11**
**CYP88D4**	**LotjaGi3g1v0339300**	**10.24**	**0.68**	**0.091**
CYP88D5	LotjaGi3g1v0338600	−0.20	−2.29	0.695
CYP71D353	LotjaGi3g1v0338500	3.68	−0.03	1.000
Transcription factor (clade Ia bHLH)				
**bHLH50**	**LotjaGi1g1v0340900**	**8.57**	**1.47**	**7.6E−13**
**clade Ia bHLH**	**LotjaGi1g1v0141500**	**8.58**	**0.71**	**0.00102**
**clade Ia bHLH**	**LotjaGi1g1v0775300**	**8.90**	**1.70**	**3.9E−12**
clade Ia bHLH	LotjaGi5g1v0218800	5.46	0.82	1
clade Ia bHLH	LotjaGi2g1v0333800	−2.75	−2.85	0.246
clade Ia bHLH	LotjaGi3g1v0404200	3.53	0.03	1
FAMA (clade Ia bHLH)	LotjaGi2g1v0285100	N. D.	—	—
MUTE (clade Ia bHLH)	LotjaGi6g1v0298200	−2.74	0.13	1
SPCH (clade Ia bHLH)	LotjaGi1g1v0281900	−2.74	−3.17	0.3

The differentially expressed genes (DEGs) are highlighted in bold. Positive log_2_ (fold change) values indicated that the genes were expressed at higher levels in the BpbHLH9-expressing lines than in the GFP-expressing control lines. Expression level was normalized with trimmed mean of M values (TMM) method. To identify DEGs, the false discovery ratio (FDR) threshold was set to ≤0.1. bAS, β-amyrin synthase; LUS, lupeol synthase; aAS, α-amyrin synthase; CAS, cycloartenol synthase; LAS, lanosterol synthase; CSyGT, cellulose synthase-derived glycosyl transferase; DDMPT, 2,3-Dihydro-2,5-dihydroxy-6-methyl-4*H*-pyran-4-one transferase; mix AS, mix amyrin synthase.

In the present study, although we introduced *BpbHLH9* expression cassette instead of *LjbHLH50* into *L. japonicus* hairy roots, *LjbHLH50* expression was upregulated (Table 1). To exclude the possibility that the sequencing reads of *BpbHLH9* were mismapped to the *LjbHLH50* locus during mapping, we visualized the mapped reads using Integrative Genomics Viewer ver. 2.13.2 (Thorvaldsdóttir et al. 2013). Even though N-terminal (5′)-side sequences were not conserved between these two genes (Supplementary Figure S3A), sequencing reads were mapped throughout the *LjbHLH50* locus (LotjaGi1g1v0340900, Supplementary Figure S3B). This observation indicated that BpbHLH9 upregulated *LjbHLH50* expression, although the detailed molecular mechanism is unclear, suggesting the presence of a self-activating loop mechanism by the bHLHs to maintain the expression of BA biosynthetic genes. Similar self-activating loops have been proposed to regulate plant secondary metabolite biosynthesis, including anthocyanins and monoterpene indole alkaloid biosynthesis (Baudry et al. 2006; Van Moerkercke et al. 2016). These mechanisms are likely important for reaching the appropriate expression levels in response to stimulation (Bateman 1998). *L. japonicus* genome contains nine clade Ia members, including LjbHLH50. BpbHLH9 expression significantly upregulated the expression of two LjbHLH50 paralogs (58.1% and 47.2% amino acid sequence identity to LjbHLH50) that were the closest to LjbHLH50 in the phylogenetic tree (Supplementary Figure S1; Table 1).

This self-activating loop is likely specific to BpbHLH9 because no clade Ia bHLHs were upregulated by *LjbHLH50* expression (Supplementary Table S5; Suzuki et al. 2022). To check if LjbHLH50 activates the expression of itself, we performed quantitative PCR (qPCR) experiments on *GFP-*expressing and *LjbHLH50*-expressing hairy root lines following our previous study (Suzuki et al. 2022). Primer sets were designed to amplify 5′ unique sequences of exogenous and endogenous *LjbHLH50*, respectively (Supplementary Table S2). While specific expression of exogenous *LjbHLH50* was observed in *LjbHLH50*-expressing lines, endogenous *LjbHLH50* expression was not significantly affected (Supplementary Figure S4). These results indicated that the functions of LjbHLH50 and BpbHLH9 are not completely the same and that LjbHLH50 is not a hub of the self-activating loop of clade Ia bHLHs. Other clade Ia bHLHs may be responsible for this self-activating loop to maintain the expression level of BA biosynthetic genes in *L. japonicus*. These findings shed light on the intricate regulatory mechanisms governing the production of bioactive C-28-oxidized triterpenes and provide a foundation for future research aimed at enhancing the production of these valuable compounds in plant hosts.

## Abbreviations

BA: betulinic acid
bHLH: basic helix-loop-helix
DEG: differentially expressed gene
GC-MS: gas chromatography-mass spectrometry
OA: oleanolic acid
OSC: oxidosqualene cyclase
P450: cytochrome P450 monooxygenase
RNA-seq: RNA-sequencing
SB: soyasapogenol B
TF: transcription factor

## Description of Supplementary Files

Supplementary Figure S1. Phylogenetic tree of clade Ia bHLH proteins.

Supplementary Figure S2. RNA-seq analysis of GFP- and BpbHLH9-expressing hairy root lines.

Supplementary Figure S3. Mapping of sequencing reads on *LjbHLH50* genomic locus.

Supplementary Figure S4. Quantitative reverse transcription-PCR (qRT-PCR) analysis of exogenous and endogenous *LjbHLH50* in *LjbHLH50*-overexpressing hairy roots.

Supplementary Table S1. Synthesized BpbHLH9 gene fragments.

Supplementary Table S2. List of primers.

Supplementary Table S3. List of fastq files.

Supplementary Table S4. List of differentially expressed genes (DEGs) identified in this study.

Supplementary Table S5. List of differentially expressed genes (DEGs) common in *LjbHLH50* and *BpbHLH9*-expressing hairy roots.
