# Supplementary material for: Ectopic expression of BpbHLH9 suggested the presence of a self-activating loop mechanism of clade Ia bHLHs to enhance betulinic acid biosynthesis in Lotus japonicus hairy roots
Source: Plant Biotechnol (Tokyo). 2024 Sep 25;41(3):319–23. doi: 10.5511/plantbiotechnology.24.0717b (PMC11962628; doi:10.5511/plantbiotechnology.24.0717b)
Supplement: Supplementary Data [file plantbiotechnology-41-3-24.0717b-s001.pdf]

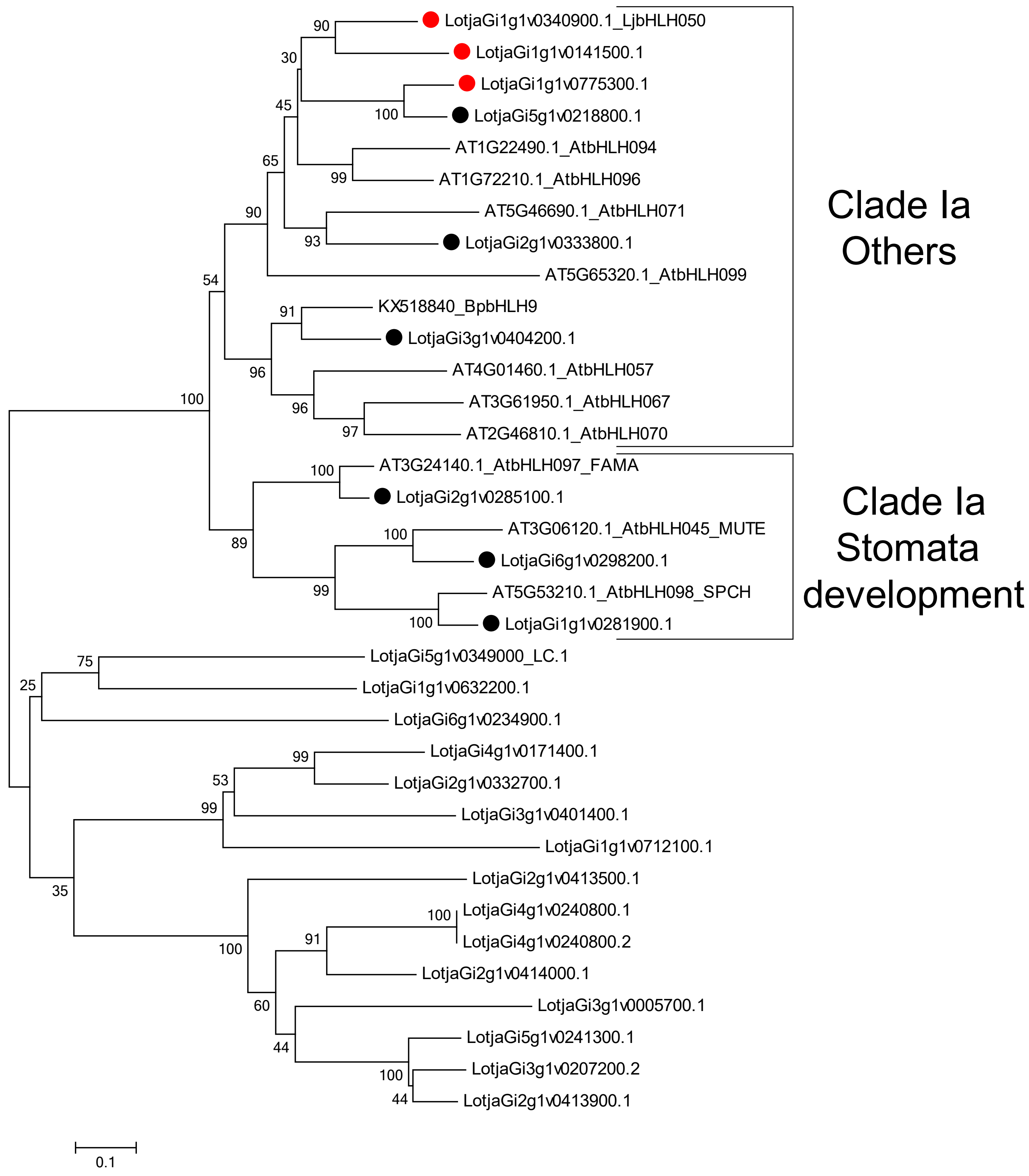

Supplementary Figure S1. Phylogenetic tree of clade Ia bHLH proteins. Circles indicate the clade Ia bHLHs in *L. japonicus*. Red circles correspond to the bHLHs whose expression was upregulated by ectopic BpbHLH9 expression in *L. japonicus* hairy roots. Protein sequences were aligned using MUSCLE (Edgar 2004). A phylogenetic tree was constructed by the neighbor-joining method with 1000 replicates using MEGA6 software (Tamura et al. 2013).

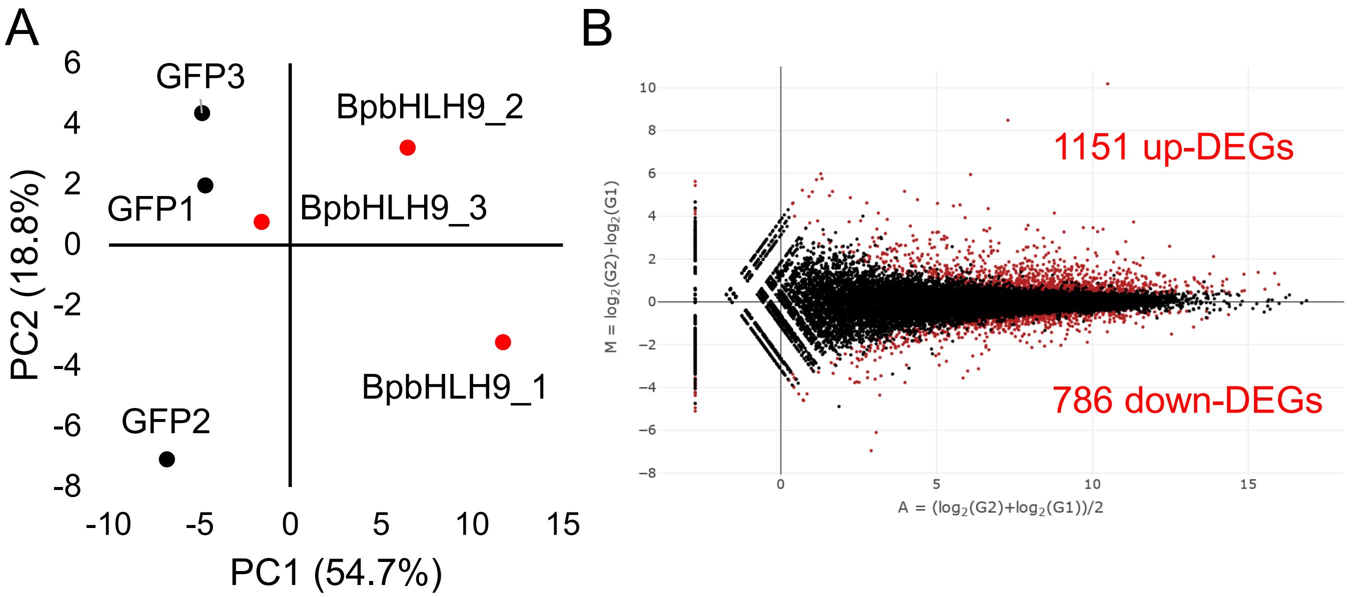

Supplementary Figure S2. RNA-seq analysis of GFP- and BpbHLH9-expressing hairy root lines. (A) Principal component analysis based on transcriptomic data. (B) MA plots to identify differentially expressed genes (DEGs). Group 1 (G1) and G2 are GFP- and BpbHLH9-expressing lines, respectively. X axis indicates A value, $\log_2$ (mean expression level). Y axis means M value, $\log_2$ (fold change of gene expression between G1 and G2). Red plots are DEGs. The threshold of the false discovery rate (FDR) is ≤ 0.10.

A

```
Score           Expect  Method                          Identities     Positives      Gaps
204 bits(519)   6e-68   Compositional matrix adjust.    108/208(52%)   145/208(69%)   24/208(11%)

Query  111  NMEEIENQRMTHIAVERNRRKQMNEYLSALRSLMPSSYVQRGDQASIIGGAINFVKELEQ  170
            N EE+E+QRMTHIAVERNRR+QMN++L+ L+SLMP+SY+QRGDQASIIGGAI+FVKELEQ
Sbjct  173  NKEEVESQRMTHIAVERNRRRQMNDHLNVLKSLMPASYIQRGDQASIIGGAIDFVKELEQ  232

Query  171  LLQSMEGQKKTDQ-----PGSSPLPPFAE-----FFVFPQYTTRAATHSNDTNMG-----  215
            LL S+E +K+  +      GSS +            FF+ PQ T       S + N G
Sbjct  233  LLHSLEAKKRMTKNQEAGDGSSSVSGAVAVSSTGFFISPQCTVG----SEEGNYGEEVKA  288

Query  216  -----VADIEVTLVDSHANLKILSKKRRGNLMKIIGGIQSLSLTILHLNVTTVDDLVLYS  270
                 VADIEVT++ +H NLKI  ++R G L+K I   ++ L LT+LHLN+T+ D  VLYS
Sbjct  289  ENKSEVADIEVTVIQTHVNLKIQCRRRPGLLLKAIVALEDLRLTVLHLNITSSDSSVLYS  348

Query  271  VSLKVEEECQLSTVDEIAAAVNQLLSMV  298
             +LK+E+EC+L + DEIA+ V+Q+LS +
Sbjct  349  FNLKIEDECKLGSADEIASTVHQILSFI  376
```

B

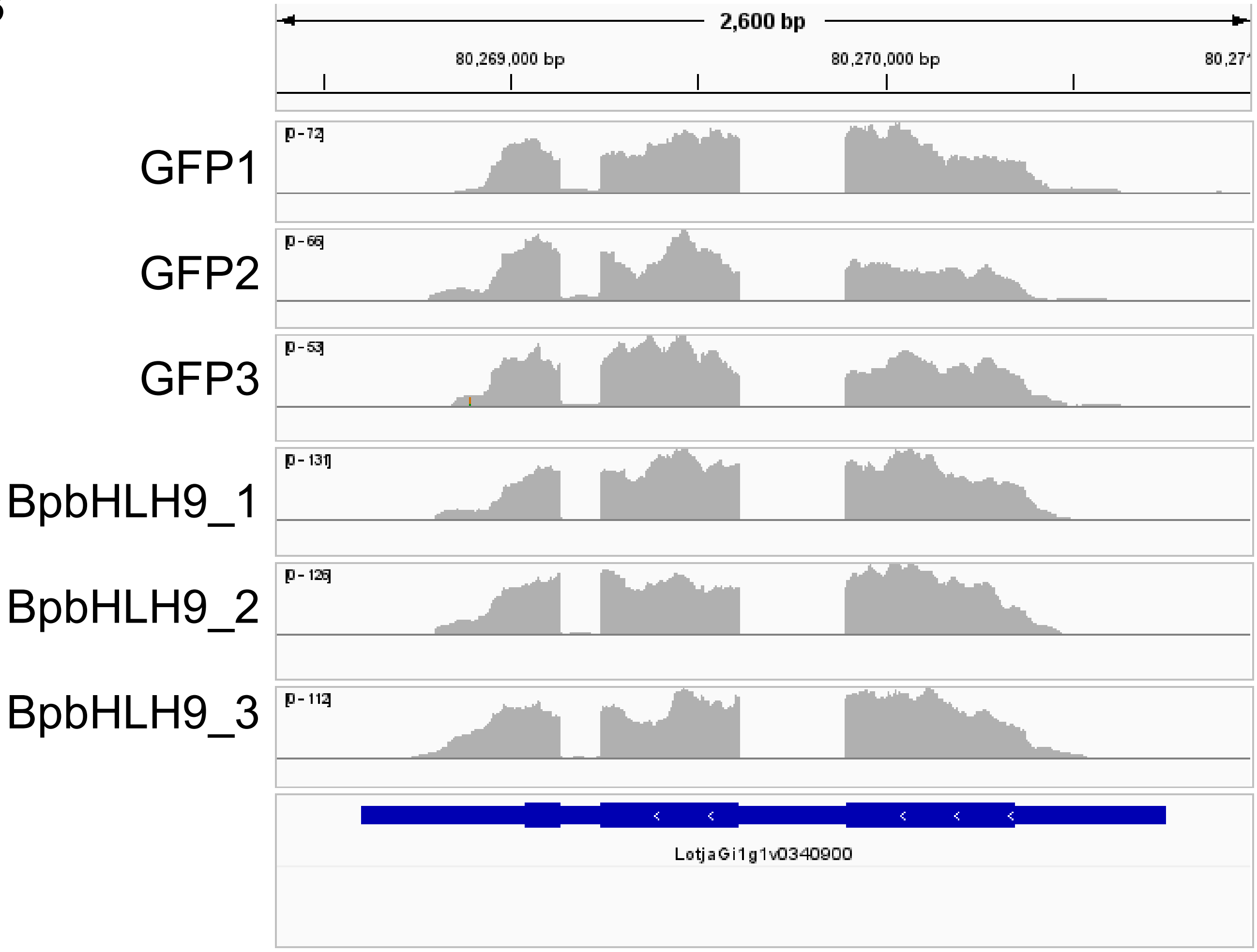

Supplementary Figure S3. Mapping of sequencing reads on *LjbHLH50* genomic locus. Upper panel is amino acid sequence alignments of LjbHLH50 (Query) and BpbHLH9 (Sbjct). Lower panel is mapping of RNA-sequencing reads of GFP- and BpbHLH-expressing hairy root lines on *LjbHLH50* genomic locus (LotjaGi1g1v0340900) visualized by Integrative Genomics Viewer.

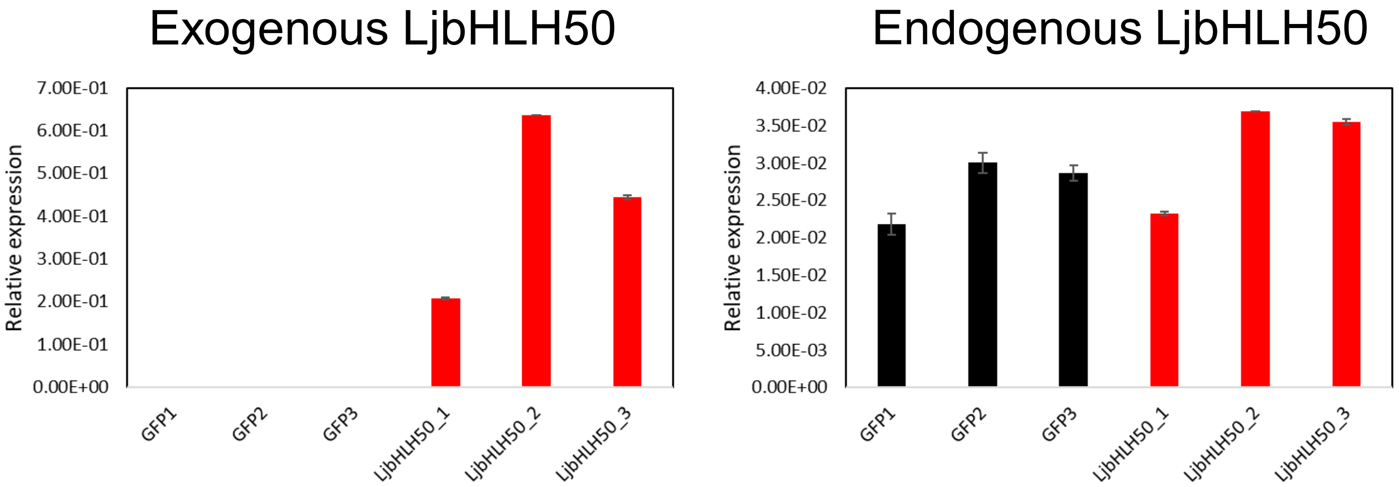

Supplementary Figure S4. Quantitative reverse transcription-PCR (qRT-PCR) analysis of exogenous and endogenous *LjbHLH50* in *LjbHLH50*-overexpressing hairy roots. Primer sets specific to endogenous *LjbHLH50* and *LjbHLH50* expression cassette in T-DNA region were used. The values were normalized to the expression level of a reference gene (*LjUBQ1*). Means ± standard deviations (SDs).

References for supplementary data

Edgar RC (2004) MUSCLE: multiple sequence alignment with high accuracy and high throughput. *Nucleic Acids Res* 32: 1792–1797

Tamura K, Stecher G, Peterson D, Filipski A, Kumar S (2013) MEGA6: molecular evolutionary genetics analysis version 6.0. *Mol Biol Evol* 30:2725–2729
